# Analytical Calculation of Relationship Temperature and Fatigue and Creep Strength Based on Thermal Activation

**DOI:** 10.3390/ma17133055

**Published:** 2024-06-21

**Authors:** Keiji Houjou, Kazumasa Shimamoto, Haruhisa Akiyama, Yu Sekiguchi, Chiaki Sato

**Affiliations:** 1 Nanomaterials Research Institute, The National Institute of Advanced Industrial Science and Technology, Tsukuba 305-8564, Japan; kazumasa.shimamoto@aist.go.jp (K.S.); h.akiyama@aist.go.jp (H.A.); 2 Precision and Intelligence Laboratory, Tokyo Institute of Technology, Yokohama 226-8501, Japan; sekiguchi.y.aa@m.titech.ac.jp (Y.S.); csato@pi.titech.ac.jp (C.S.)

**Keywords:** epoxy adhesive, lap joint, fatigue strength, creep strength, temperature dependence

## Abstract

The purpose of this study was to formulate a mathematical expression for the temperature dependence of adhesive strength using various parameters. Adhesive structures are typically exposed to a broad temperature range, spanning from low to high temperatures; therefore, understanding how their strength depends on temperature is crucial. The strength was measured through tensile, fatigue, and creep tests at temperatures ranging from −60 °C to 135 °C. The properties of these test types were thoroughly investigated by analyzing the strength of the test results from a thermal activity perspective. The results demonstrate that there is a clear relationship between temperature and strength. The intensity decreased with temperature according to the exponential function and could be accurately represented using the parameters of thermal activity. The temperature at which the strength begins to decrease in the fatigue test was higher than in the static tests. Consequently, we were able to accurately express the relationship between the temperature and intensity using certain parameters. Few studies successfully developed a precise nonlinear relationship between temperature and intensity using approximate expressions.

## 1. Introduction

In recent years, the demand for lightweight transportation equipment has significantly increased, driven by concerns related to environmental protection and energy conservation. One of the most effective methods for achieving lightweighting is adhesive bonding, which enables the fabrication of dissimilar material joints. Automobiles are produced in a variety of ways to reduce energy consumption [1]. In particular, weight reduction of bodies is essential to reduce energy consumption. For example, structural design innovations and improvements in steel materials were made. However, the use of lightweight materials is the most effective way to significantly reduce weight, and the development of adhesive bonding technology is rapidly advancing for this purpose [2]. To apply adhesive bonding technology to the structural components of transportation equipment, ensuring durability and reliability against environmental loads is essential. In addition to weather resistance against factors such as temperature, humidity, ultraviolet rays, salt, and acid rain, load durability must be studied simultaneously [3,4,5,6,7,8,9]. In this study, we focused on the relationship between environmental temperature and adhesive strength, which is a key factor affecting adhesive joints. The purpose of this study was to elucidate and mathematically express the relationship between environmental temperature and adhesive joint strength, thus allowing the temperature dependence of strength to be expressed objectively. Banea MD et al. [10] experimented in detail on the relationship among temperature, strength, and fracture toughness of epoxy adhesives, which was based on mode1. Tests were conducted at temperatures from *R*.*T*. to 200 °C. The strength at temperatures above the *T*_g_ (glass transition temperature) point was revealed. JINGXIN NA et al. [11] measured the static strength of polyurethane from −40 °C to 90 °C using butt joints and a single lap join and obtained an approximate linear equation between temperature and strength. However, the relationship among fatigue, creep strength, and temperature was not experimented with. Moreover, they did not generalize the formula. Therefore, we conducted a study to accurately represent the nonlinear relationship between temperature and intensity. Various studies have explored the temperature dependence of material strength and adhesive strength [10,11,12,13,14,15], demonstrating that the relationship between the two can be expressed as a formula [16,17,18]. Williams et al. [19] proposed a method (WLF, Eq.) to express the relationship between the temperature and time of strain in polymer materials. Since then, numerous research examples using the time–temperature conversion rule have been published [20,21,22]. However, in this study, for adhesive joints, the strength was tested together with the metal or resin adherend. Consequently, creating a master curve based on the strain during the test was not feasible.

In this study, the definition of the relationship between temperature and strength was investigated based on the hypothesis that strength reduction depends on the increase in the number of defects due to heat. This study aimed to establish the relationship between temperature and intensity.

## 2. Specimen and Experimental Procedure

### 2.1. Adhesive

A thermoset epoxy adhesive supplied by Cemedine Co., Ltd., Koga, Japan, was selected for this study. Its chemical composition is listed in Table 1. Carboxyl-terminated butadiene-nitrile rubber (CTBN) was added to the adhesive to improve toughness and elongation at fracture.

### 2.2. Test Specimen

**Figure 1:**
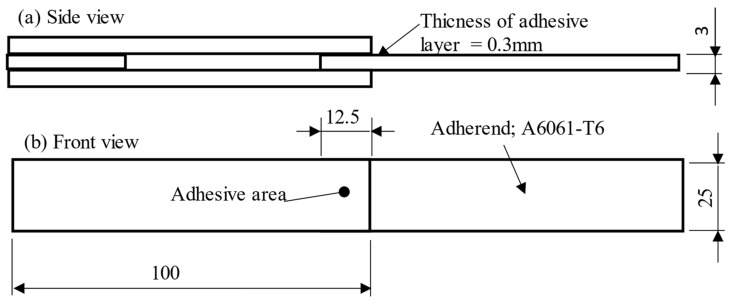
Schematic and dimensions of test specimen used in this study: (**a**) side view; (**b**) front view.

Figure 1 shows the test specimens used in this study, consisting of A6061-T6 aluminum plates with a width of 25 mm and thickness of 3 mm, which served as adherends. Prior to assembly, the adherends underwent a surface treatment process, including degreasing with acetone, followed by alkaline and acid cleaning in a hot bath at 60 °C for 30 s. Afterward, the adhesive was applied to the aluminum adherends and cured at 180 °C for 1 h, ensuring a controlled adhesive layer thickness of 0.3 mm.

### 2.3. Experimental Procedure

The experiments included tensile shear, fatigue, and creep tests. The atmospheric temperature was measured stepwise from −60 °C to 135 °C in an electric furnace. However, humidity was not controlled. The test conditions were as follows: the tensile test rate was 1 mm/min, the fatigue test was conducted at a frequency *f* = 10 Hz, with a stress ratio *R* = −1, and the creep test was conducted under load control. If the specimen did not fracture within a certain time or number of times, the maximum stress was defined as the creep or fatigue limit. Tensile and creep tests were performed using a tensile testing machine (RTF-1350 Tensilon, A&D Corporation, Kitamoto, Japan) with an electric furnace and a maximum load of 50 KN. The control temperature of the electric furnace ranged from −45 °C to 210 °C in air. A hydraulic-type fatigue testing machine (EHF-E50KN Servo Pulser, Shimadzu Corporation, Kyoto, Japan) with an electric furnace was used. The control temperature of the electric furnace ranged from −60 °C to 250 °C in air. The clamps were hydraulically operated, and a maximum cyclic load of 50 KN was applied. The fracture surfaces of the specimens were observed using an optical microscope, and the chemical structure of the adhesive was investigated using Fourier transform infrared (FTIR) spectroscopy.

## 3. Results and Discussion

### 3.1. Relationship between Test Temperature and Shear Strength

**Figure 2:**
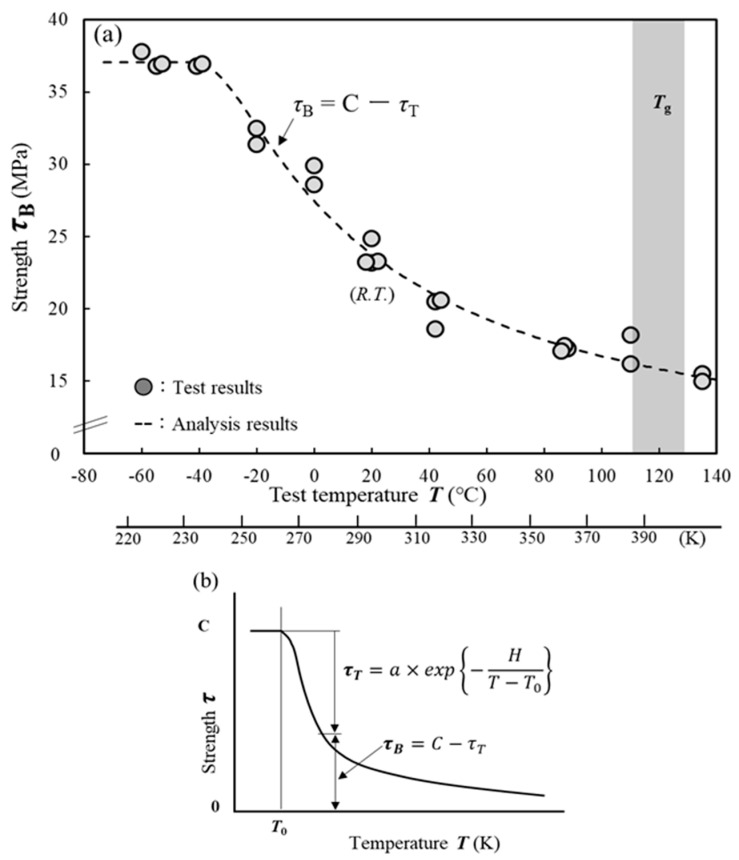
Effect of temperature on shear strength. (**a**) Relationship between test temperature *T* and shear strength *τ*_B_. (**b**) Schematic showing the relationship between temperature and strength.

Figure 2a shows the relationship between test temperatures ranging from −60 °C to 135 °C and the shear strength of adhesive joints. The strength decreased as the test temperature increased and improved as the test temperature decreased; however, it did not exhibit further increases below a certain temperature. In all the cases, cohesive failure was observed as the failure mode. However, the fracture surface tested below 0 °C was a brittle cleavage fracture surface accompanied by thin-layer cohesive failure. As the temperature increased, the fracture changed to ductile fracture, accompanied by elongation. The gray shaded area in Figure 2a represents the *T*_g_ of the adhesive. Table 2 lists the bulk mechanical properties and *T*_g_ points measured in previous studies. The Young’s modulus, Poisson’s ratio, and tensile strength of the bulk specimens were 1100 MPa, 0.41, and 27 MPa at *R. T*., respectively. *T*_g_ was 110–125 °C [7,9]. The adhesive strength gradually decreased up to 135 °C, and a significant drop in strength was not observed even above *T*_g_.

In prior research, the authors of the current study developed a mathematical relationship between test temperature *T* and shear strength *τ*_B_ within a range from −20 °C to 135 °C, as shown in Equation (1) [15]. Figure 2b shows the relationship between temperature and strength reduction schematically. The proposal suggested that the strength *τ*_B_ at the test temperature *T* could be calculated by subtracting the amount of strength reduction *τ*_T_ owing to the test temperature from the strength *C* [15]. However, various studies have demonstrated the existence of a maximum temperature *T*_0_ at which the strength does not decrease [12,16,18,23,24,25]. Therefore, the equation was modified; when *T* < *T*_0_, Equation (2) was used in this study.

(1) $${T\geq T_{0}\;\;\;\;\tau }_{B}=C-a\cdot exp\left\{-\frac{H}{T-T_{0}}\right\}$$

(2) $${T_{0}>T\;\;\;\;\;\;\;\;\;\;\;\;\tau }_{B}=C$$

where *C* represents the original strength of the material before it decreases with temperature, *a* represents a proportionality constant that converts the probability of the existence of defects into material strength, and *H* includes the Boltzmann constant and activation energy. The constants are discussed later in Section 3 and Section 4. However, at low temperatures, it is influenced by the difference in the linear expansion coefficient between the adherend and adhesive [25]. Therefore, the applicable lower-limit temperature of Equation (2) is unknown. The dashed line in Figure 2a represents the curve generated by substituting the intensity data obtained in the experiment into Equations (1) and (2) and determining the constants *C*, *a*, *H*, and *T*_0_ using the least-squares method.

### 3.2. Relationship between Test Temperature and Fatigue Strength

**Figure 3:**
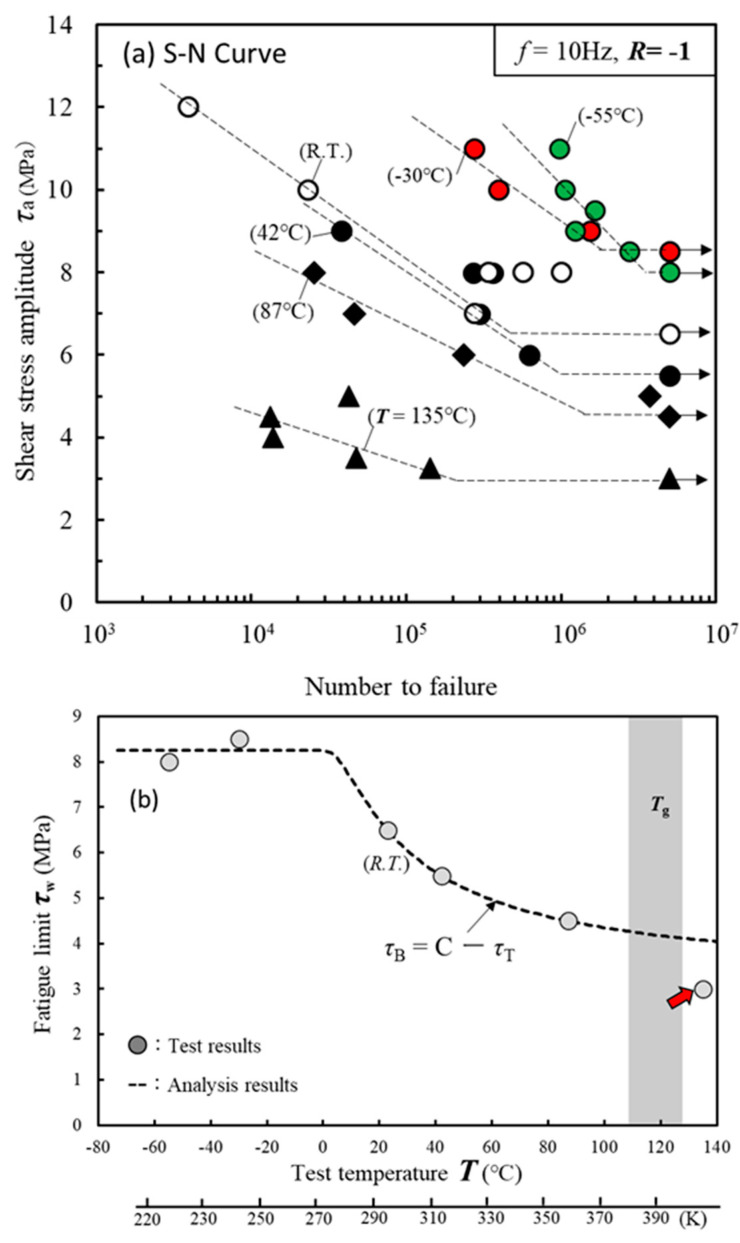
Effect of test temperature on fatigue strength. (**a**) S-N curve from −55 °C to 135 °C. (**b**) Relationship between test temperature *T* and fatigue limit *τ*_w_.

We then studied whether Equations (1) and (2) could be applied to the relationship between the test temperature and fatigue limit. Figure 3a shows the fatigue test results from −55 °C to 135 °C. The frequency and stress ratios were *f* = 10 Hz and *R* = −1. The tests were terminated when the specimens did not fail until *N* = 5 × 10^6^. The maximum amplitude stress *τ*_a_ was defined as the fatigue limit *τ*_w_. The arrows indicate tests that were terminated without failure. The fatigue limit improved significantly as the test temperature *T* decreased. Figure 3b represents the relationship between test temperature *T* and fatigue limit *τ*_w_. The dashed line is an approximation line calculated by substituting the test results into Equations (1) and (2).

**Figure 4:**
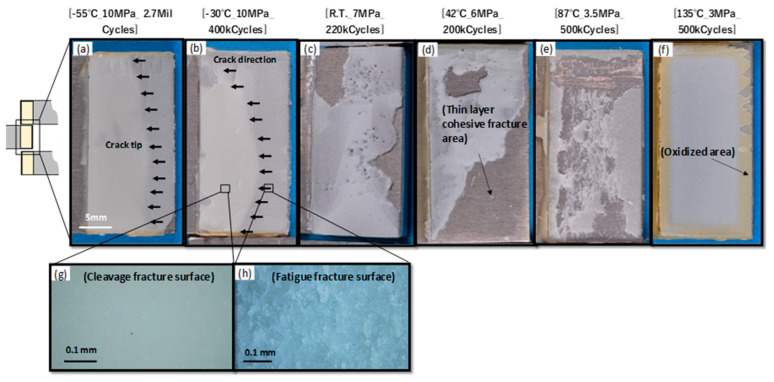
Photographs of fractured surfaces during fatigue tests at various temperatures. (**a**–**f**) Fracture surface under each test condition. (**g**,**h**) Details of fatigue fracture surface.

This approximation line closely matches the experimental results. However, the fatigue limit of 135 °C (red arrow) is out of the approximate line. As explained later in Figure 4, at 135 °C, Equation (1) was not able to approximate the experimental results because the oxidation or hydrolysis of the adhesive contributed to reducing the strength, in addition to *τ*_T_. Figure 4 shows photographs of the fracture surface at each temperature. All fracture surfaces exhibited cohesive failure. Extremely thin adhesive layers remained on the opposite adherent surfaces. The test temperatures in Figure 4a,b are −55 °C and −30 °C, respectively, and fatigue fracture surfaces, indicated by the arrows, can be observed. Figure 4h shows a detailed view of the fatigue-fractured surface with wave patterns perpendicular to the crack propagation direction. In contrast, in Figure 4g, a brittle fracture surface can be observed, indicating an unstable fracture. Figure 4c–e show ductile cohesive failures of the adhesive. In particular, Figure 4e shows that failure occurred deep within the adhesive layer. A discolored oxidized area with reduced strength is visible within a width of 3 mm from the edge.

### 3.3. Relationship between Test Temperature and Creep Strength

**Figure 5:**
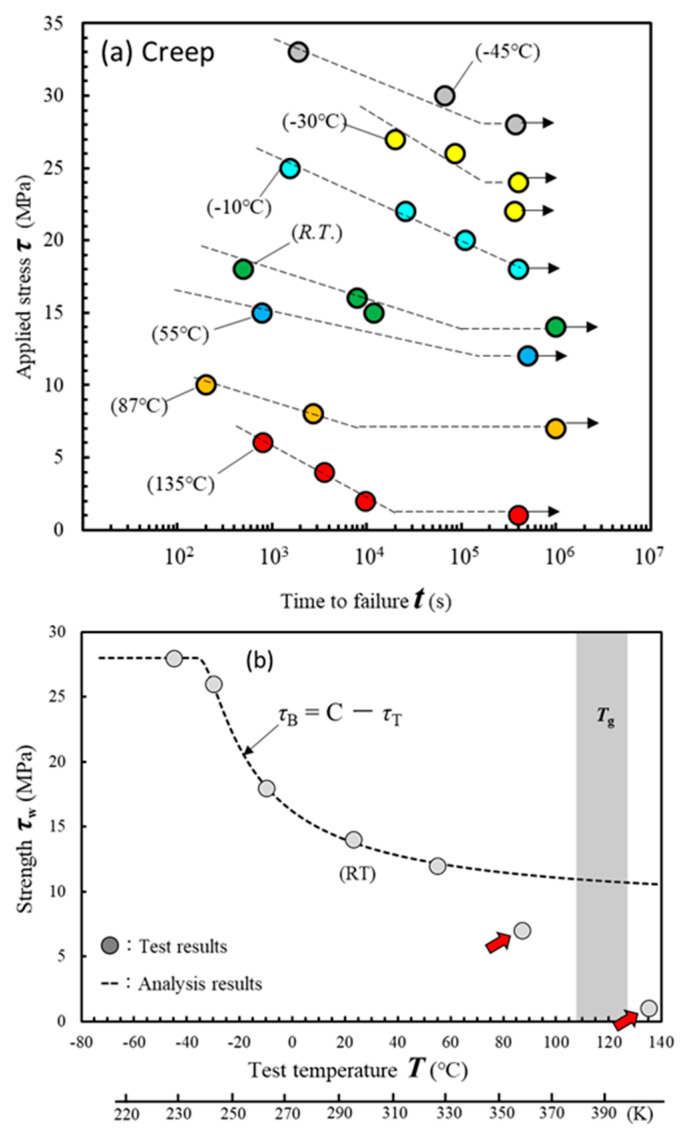
Effect of test temperature on creep strength. (**a**) Relationship between test temperature *T* and creep limit *τ*_w_. (**b**) Schematic showing the relationship between temperature and strength.

We then studied whether Equations (1) and (2) could be applied to the relationship between the test temperature and creep limit. Figure 5a shows the creep test results from *T* = −45 °C to 135 °C. The creep limit *τ*_w_ was defined as the maximum stress *τ* that did not fail for over 400,000 s. The arrows in the figure indicate that the tests were terminated without fracture. The range of fatigue limit *τ*_w_ was 5.5 MPa (8.5 MPa to 3 MPa, Figure 3a); however, that of creep limit *τ*_w_ was 27 MPa (28 MPa to 1 MPa, Figure 5a). The creep strength was sensitive to the test temperature.

Figure 5b shows the relationship between test temperature *T* and creep limit *τ*_w_. The dashed line is an approximation calculated by substituting the test results into Equations (1) and (2). This approximation line closely matches the experimental results. For the same reason as observed in the fatigue test, the creep limits of 87 °C and 135 °C (red arrows) are also out of the approximate line. Because the exposure time of the creep tests were longer than that of the fatigue tests, the deterioration of the adhesive progressed significantly during the high-temperature test, resulting in a decrease in strength.

**Figure 6:**
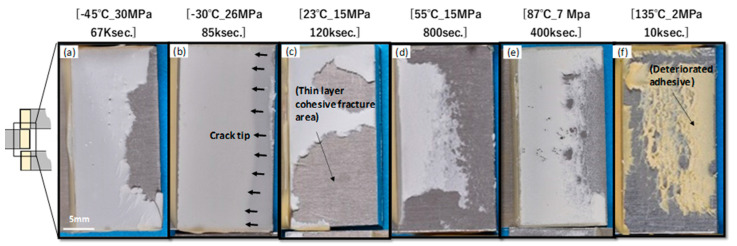
Photographs of fractured surfaces during creep tests at various temperatures. (**a**–**f**) Fracture surface under each test condition.

Figure 6 shows photographs of the fractured surfaces during testing. These fracture surfaces exhibited cohesive or thin-layer cohesive failures. As shown in Figure 6a,b, the adhesive exhibits brittle fracture behavior at low temperatures. The arrows indicate the crack tip at the time of fracture. When the test temperature exceeded *R.T*. (controlled to 23–28 °C), cohesive failure transitioned to ductile failure, as shown in Figure 6c–f. Cohesive failure occurred deep within the adhesive as the test temperature increased. In the test at 135 °C, as shown in Figure 5f, the adhesive color changed, indicating deterioration due to oxidation. Figure 5b shows that the strengths at 87 °C and 135 °C are significantly lower than the values calculated from Equations (1) and (2) (dashed line).

**Figure 7:**
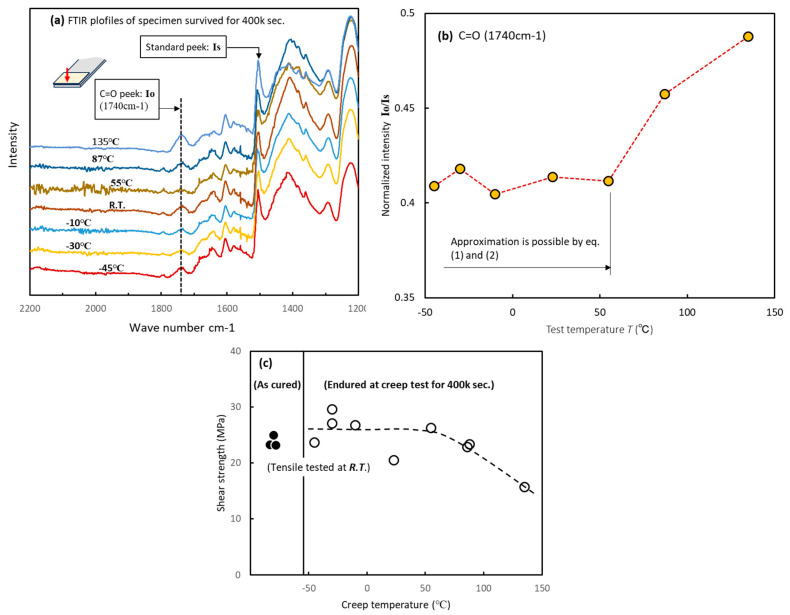
Degradation behavior of adhesives during creep tests. (**a**) FTIR profiles of specimens that survived for four million seconds. (**b**) Relationship between test temperature and intensity of C=O peaks. (**c**) Relationship between test temperature and shear strength of specimens that endured creep tests for 400,000 s.

FTIR measurements were performed to investigate the deterioration of the adhesive. Specimens that endured for 400,000 s were then subjected to fracture testing. Subsequently, FTIR measurements were taken at a location 2–3 mm from the edge of the adhesive. Figure 7a shows the FTIR profiles of the adhesive that underwent the creep test from −45 °C to 135 °C for 400,000 s. The peak at 1740 cm^−1^ (*I*o) indicates a C=O bond. *I*o is the peak generated by the oxidation or hydrolysis of adhesives in a high-temperature atmosphere and can be considered a barometer for adhesive deterioration [26]. The *I*o peak at 135 °C clearly increases. The *I*o peaks were extracted from the profile and normalized using the intensity (*I*s) of the fingerprint area peak at 1505 cm^−1^, and the (*I*o/*I*s) is shown in Figure 7b. The C=O peak did not change significantly from −45 °C to 55 °C but exhibited a notable increase above 87 °C. Consequently, the creep strength decreased in Figure 5b. Therefore, in conjunction with Figure 5b, these results suggest that, if oxidation and hydrolysis do not progress significantly, the creep strength can be explained by Equations (1) and (2).

Figure 7c shows the results of measuring the *R.T*. shear strength of the specimens that did not fail during creep tests. A clear decrease in shear strength was not observed from −55 °C to 54 °C, but the shear strength decreased above 87 °C. These findings alongside the results in Figure 7b suggest that the adhesive maintained sound strength and chemical structure at test temperatures up to 55 °C.

### 3.4. Temperature Dependence of LJ Strength

**Figure 8:**
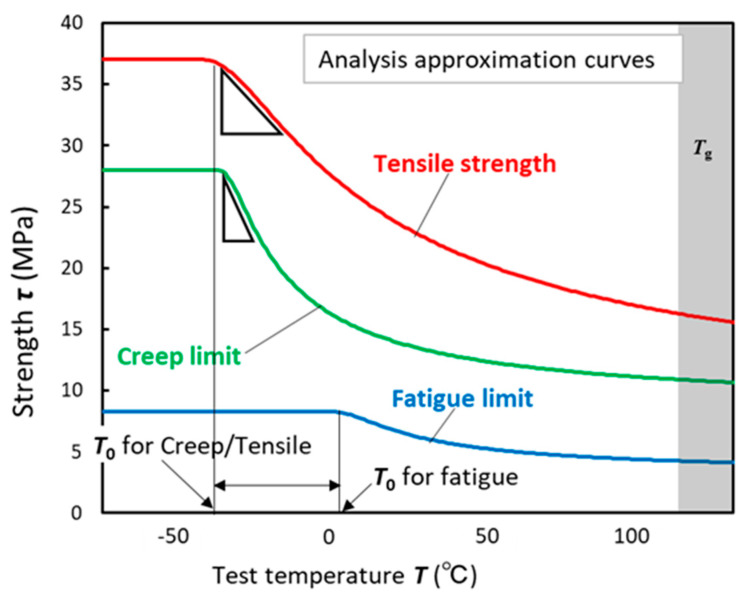
Comparison of approximate lines for tensile, fatigue, and creep tests.

The temperature-dependent properties for each test type were studied based on the approximation lines of the tensile, fatigue, and creep tests. Figure 8 shows the approximation lines for the three tests used in this study. Some of these trends are described below.

*T*_0_ of the fatigue test was considerably higher than those of the tensile and creep tests. In Equation (1), the assumption is that the strength decrease depends on the increase in defects owing to temperature (thermal activation and thermal vibration). However, the temperature at which defects begin to be produced owing to thermal vibration is not necessarily at the same temperature. Therefore, the strength did not decrease until the temperature reached the point at which the heat began to exert an influence in the case of creep and tensile tests. However, in the fatigue tests, cyclic strain was added to the effects of thermal vibration. Consequently, the specimens were in a high-energy state, causing the strength to begin decreasing at higher temperatures compared to the creep and tensile tests. As shown in Table 3, the fatigue *T*_0_ is 40 to 50 °C higher than the *T*_0_ for the other tests. Another characteristic of Figure 8 is the high sensitivity of the creep strength to the test temperature. The triangle mark in the figure indicates that the slope of the strength in the creep test was steeper than that in the other tests. As indicated in Table 3, constant *H*, which depends on activation energy, was the lowest during the creep test. This corresponds directly to the shape of the curve shown in Figure 8. Based on the aforementioned results, we developed a method to describe the temperature dependence of the strength using the parameters *T*_0_ and *H*.

## 4. Conclusions

A detailed investigation of tensile, fatigue, and creep strengths in epoxy adhesive joints across a range of temperatures was conducted. Consequently, we reached the following conclusions:

(1) Tensile strength, fatigue limit, and creep limit improved with lower test temperatures; however, no improvement occurred below a certain temperature.
(2) The relationship between test temperature and strength can be approximated using a thermal activation equation.
(3) In long-term high-temperature tests, such as 135 °C fatigue tests, the adhesive deteriorates and does not follow the established curve.
(4) Observations of the fracture surface in the fatigue test revealed that brittle fracture occurred when the test temperature was low, whereas ductile fracture occurred when it was high.
(5) The *T*_0_ point for fatigue was higher than those of the other tests, and the temperature sensitivity of strength, represented by *H*, was highest in the creep test.
(6) A method to express the temperature dependence of adhesive strength using constants *T*_0_ and *H* was proposed.

In this study, we conducted experiments using assembled lap joints. However, it is desirable to conduct clear research using only adhesive materials. Furthermore, adaptation of Equations (1) and (2) to metallic and inorganic materials as well as appreciation of statistical analysis for fatigue and creep test results are a future study.

## Figures and Tables

**Table 1:** Chemical composition of adhesive (mass%).

Material	Mass %
Bisphenol A epoxy resin	24
CTBN-modified epoxy resin (elastomer 40%) CTBN; carboxyl-terminated butadiene acrylonitrile rubber	39
Fumed silica	3
Filler (CaCO_3_)	26
CaO	2
Dicyane diamide	5
3-(3,4-dichlorophenyl)-1,1′-dimethylurea	1

**Table 2:** Mechanical properties of cured adhesive.

Bulk Mechanical Properties [7,9]	
Tensile strength at R.T. (MPa)	30
Young’s modulus at R.T. (MPa)	1100
Poisson’s ratio at R.T.	0.41
*T*_g_ point (°C)	110–125

**Table 3:** Constants *T*_0_ and *H* calculated from experimental results using the least-squares method.

Test Type	*T*_0_ (℃)	*H* (*Q*/*B*) (×10^3^K/mol)
Tensile	−49	54
Fatigue	−3	28
Creep	−39	19

## Data Availability

Data are contained within the article.
